# Stakeholder Perception of the Implementation of Genetic Risk Testing for Twelve Multifactorial Diseases

**DOI:** 10.3390/genes15010049

**Published:** 2023-12-28

**Authors:** Tomoharu Tokutomi, Akiko Yoshida, Akimune Fukushima, Fuji Nagami, Yuko Minoura, Makoto Sasaki

**Affiliations:** 1 Iwate Tohoku Medical Megabank Organization, Iwate Medical University, Shiwa 020-3694, Japan; akikoyos@iwate-med.ac.jp (A.Y.);; 2 Department of Clinical Genetics, School of Medicine, Iwate Medical University, Iwate 020-8505, Japan; 3 Tohoku Medical Megabank Organization, Tohoku University, Sendai 980-0872, Japan; 4 Departments of Medical Genetics and Genomics, School of Medicine, Sapporo Medical University, Sapporo 060-8556, Japan

**Keywords:** polygenic risk score, multifactorial disease, stakeholder perception, adult-onset disease, clinical implementation

## Abstract

Genome-wide association studies have been employed to develop numerous risk prediction models using polygenic risk scores (PRSs) for multifactorial diseases. However, healthcare providers lack confidence in their understanding of PRS risk stratification for multifactorial diseases, which underscores the need to assess the readiness of PRSs for clinical use. To address this issue, we surveyed the perceptions of healthcare providers as stakeholders in the clinical implementation of genetic-based risk prediction for multifactorial diseases. We conducted a web-based study on the need for risk prediction based on genetic information and the appropriate timing of testing for 12 multifactorial diseases. Responses were obtained from 506 stakeholders. Positive perceptions of genetic risk testing were found for adult-onset chronic diseases. As per participant opinion, testing for adult-onset diseases should be performed after the age of 20 years, whereas testing for psychiatric and allergic disorders that manifest during childhood should be performed from birth to 19 years of age. The stakeholders recognized the need for genetic risk testing for diseases that develop in adulthood, believing that the appropriate testing time is after maturity. This study contributes to the discussion on the clinical implementation of the PRS for genetic risk prediction of multifactorial diseases.

## 1. Introduction

Multiple genetic and environmental factors can cause multifactorial diseases, such as heart disease, diabetes, cancer, dementia, and depression. Owing to the high number of patients affected by these diseases and the equally high number of individuals who may potentially be affected in future, these diseases are of high importance in the framing of preventive health policies. The health policies of several countries, including Japan, are promoting measures to prevent the onset of multifactorial diseases. In addition, the World Health Organization has classified these diseases as noncommunicable diseases and formulated a global action plan for their prevention and control [1]. As highlighted in prior studies, a complete understanding of the complex interplay of genetic factors in multifactorial diseases is crucial for developing and implementing polygenic risk scores (PRSs) in clinical settings [2,3].

The genetic risk of developing a multifactorial disease can be predicted using PRSs [4,5,6,7,8]. The PRS is the weighted sum of the number of risk alleles carried by an individual. Similar to blood pressure and other biochemical markers currently used in clinical practice, the PRS is considered a novel risk prediction indicator. PRSs enable the identification of individuals at high risk of developing disease during primary care. Therefore, disease onset can be prevented at an earlier stage, improving the overall quality of life and reducing the burden on the healthcare system [9,10]. Currently, studies on the practical application of PRS-based risk prediction for multifactorial diseases are mainly conducted in Europe and the United States [11,12,13,14]. The Tohoku Medical Megabank (TMM) Project [15] conducted a study on risk assignment based on genetic information for monogenic diseases, familial hypercholesterolemia, hereditary breast and ovarian cancer syndromes, and multiple drug susceptibilities and verified the clinical usefulness and feasibility of the risk assignment system [16,17,18]. We are currently conducting a PRS-based disease risk allocation study for multifactorial diseases in a larger target population.

The clinical utility of PRSs has been established; therefore, there is a need to examine PRS-related issues, such as risk assessment, technical limitations, ethnic specificity, and the maximization of benefit to public health and individuals [9,19,20]. In addition, it is necessary to improve human resources in the implementation structure. To date, the clinical utilization of genetic information has been limited to rare diseases, such as monogenetic disorders, and to specialist medical personnel with genetic expertise. However, risk prediction for multifactorial diseases differ significantly from risk prediction for monogenetic illnesses in several vital aspects. Monogenic diseases are caused by mutations in a single gene, making their risk prediction more straightforward and often based on the presence or absence of specific mutations. In contrast, multifactorial diseases are influenced by multiple genes and environmental factors. PRSs for these diseases aggregate the effects of numerous genetic variants, each contributing a small amount to the overall disease risk. This complexity makes PRSs less deterministic and more probabilistic, providing a risk estimate rather than a definitive prediction. PRSs have the potential to make a significant impact on public health. To make this possible, PRSs need to be implemented at the population level in primary care. Target diseases include cancer and cardiovascular, neurological, and psychiatric diseases. The successful clinical implementation of genetic-based risk prediction requires the participation of multidisciplinary medical professionals who are not specialized in genetics [21,22]. For cancer and other complex diseases, an interdisciplinary working group in the U.K. and Europe recommended that medical practitioners consider implementing risk stratification based on polygenic genetic information [23]. They also proposed eight new competencies that healthcare providers will require in the future [24]. Specifically, knowledge of genetics (based on the premise that multiple genetic and environmental factors are involved in disease development), risk communication skills, and an understanding of ethical, legal, and social issues will be required. However, healthcare providers have low confidence in their knowledge of PRSs and risk stratification with regard to complex diseases, such as diabetes and heart disease [25,26,27].

Therefore, it is crucial to determine technical and social issues as well as the involvement and awareness of medical practitioners in the clinical implementation of PRSs [9,19]. In this study, we conducted a questionnaire-based survey with the aim of determining medical professionals’ understanding of the clinical application of PRSs for multifactorial diseases.

## 2. Materials and Methods

### 2.1. Participants

Overall, four types of target professionals involved in implementing risk prediction and subsequent preventive and medical interventions within the medical care framework were selected: physicians, nurses, pharmacists, and registered dieticians. The participants were recruited from 129 institutes. All institutes are members of the National Liaison Council for Clinical Sections of Medical Genetics (NLCCSMG), a network of genetic medical departments in hospitals across Japan. The recruitment method involved sending study information and pamphlets to each institute’s NLCCSMG members. Then, we called for voluntary participation from physicians, nurses, pharmacists, and registered dietitians working at each institute. The survey was conducted using a web-based multiple-choice questionnaire. Those who agreed to participate registered via a web page using their email addresses. Next, the participants read an explanation of the study and gave informed consent to participate. An invitation, containing a URL to the questionnaire, was sent via email to the registered email addresses. The survey was conducted between 29 September and 31 October 2020, using the LimeSurvey web questionnaire system form.

### 2.2. Questionnaire

The questionnaire was designed taking into consideration the report of a specialist working group that examined the challenges of implementing genome-based risk stratification screening [23,24]. The working group identified eight areas of expertise that non-genetic healthcare professionals require to implement PRSs in primary care. A unique questionnaire was created consisting of nine key areas: (1) demographics, (2) professional experience, (3) attitude toward multifactorial diseases, (4) attitude toward genetic testing for risk prediction, (5) recognition of PRSs, (6) knowledge support for healthcare providers involved in multifactorial disease risk prediction, (7) collaboration with other healthcare professionals, (8) important issues in multifactorial disease risk prediction feedback, (9) management of genetic information. The questionnaire was designed as a 5-point Likert-scale survey in a multiple-choice, multiple-answer format.

We focused on 12 diseases: diabetes, hypertension, cardiovascular disease, cerebrovascular disease, aneurysms, cancer, infectious diseases, autism spectrum disorder, depression, schizophrenia, cognitive impairment, and allergies. These diseases were identified by the Genomic Medical Conference of the Cabinet Office of Japan’s Health and Medical Strategy Promotion Council [28,29]. The questionnaire was created by specialists, including clinical geneticists and certified genetic counselors, recognized by the Japanese Society of Genetics, and approved by the advisory committee of TMM’s genetic information feedback research.

### 2.3. Statistical Analysis

The responses were rated on a 5-point Likert scale for “the necessity of genetic testing” (1 = “Agree”, 2 = “Somewhat agree”, 3 = “Neither agree nor disagree”, 4 = “Somewhat disagree”, 5 = “Disagree”) and “the appropriate age for testing”. The responses were categorized in reference to five age groups with an option for opposition to the testing (1 = “0 years old”, 2 = “1–19 years old”, 3 = “20–39 years old”, 4 = “40–59 years old”, 5 = “≥60 years old”, and “should not be performed” for those opposed to testing). The Wilcoxon rank sum test was used to compare the responses from different groups of professionals, and pairwise comparisons using the Wilcoxon rank sum test with continuity correction or pairwise comparisons of proportions were used to compare the responses received for each of the 12 diseases. Both pairwise comparisons were performed using Bonferroni correction. The significance level was set at 5% (*p* < 0.05), and two-tailed tests were used for all comparisons. All hypothesis tests were performed using R version 4.0.3.

### 2.4. Ethical Considerations

The purpose of this study and the consent items were showed to the participants before they agreed to participate. The participants had to click the “accept” button to provide consent before proceeding to the questionnaire. This study was conducted in accordance with the Declaration of Helsinki, and the protocol was approved by the Ethics Committee of the Medical Faculty of Iwate Medical University (approval no. MH2020-014).

## 3. Results

### 3.1. Participant Characteristics

A total of 539 participants (from 69 institutes affiliated with the NLCCSMG) responded. Overall, 33 answered questionnaires were excluded from the analysis because they were answered by participants who were not physicians, nurses, pharmacists, or registered dieticians. Finally, 506 answered questionnaires were included in the analysis. The characteristics of the 506 participants are presented in Table 1 and Table S1. There were 68 registered dietitians (13.4%), 112 nurses (22.1%), 107 pharmacists (21.1%), and 219 physicians (43.3%). The two age groups with the highest proportion of participants were 30–39 years and 40–49 years, which included 167 and 158 individuals, respectively (33.0% and 31.2%, respectively).

### 3.2. Awareness of Genetic Testing for Risk Prediction

**Figure 1:**
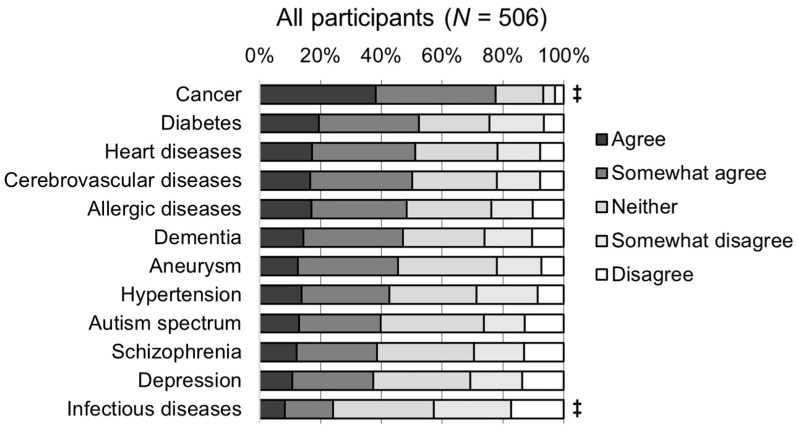
Perceptions of the need for genetic testing for multifactorial disease risk prediction. Statistical significance by pairwise comparisons using the Wilcoxon rank sum test with Bonferroni correction comparing 12 diseases: ‡ *p* < 0.001.

**Figure 2:**
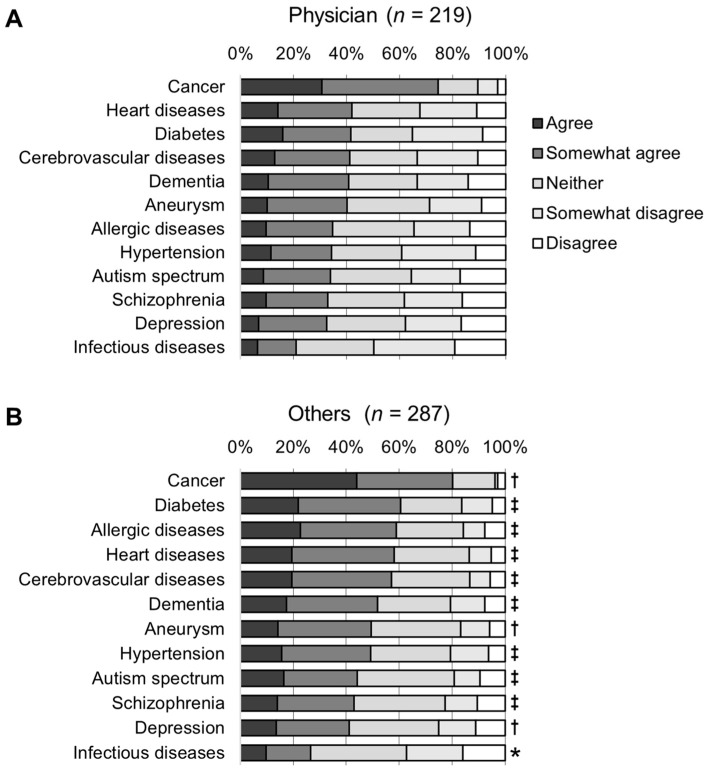
Perceptions of the need for genetic testing for multifactorial disease risk prediction. (**A**) Physicians. (**B**) Others (nurses, pharmacists, and registered dieticians). Statistical significance by Wilcoxon rank sum test compared to physicians’ perceptions: * *p* < 0.05; † *p* < 0.01; ‡ *p* < 0.001.

The results of the five-point Likert-scale survey on the necessity of genetic testing for the risk prediction of 12 diseases are shown in Figure 1. The proportion of positive opinions (agree and somewhat agree) was highest for cancer (77.7%, *p* < 0.001, Figure 1 and Table S2). That is, among the 12 diseases, the number of participants agreeing that PRSs are necessary for risk prediction was the highest for cancer. In contrast, the lowest proportion of positive opinions (24.1%) and the highest proportion of negative opinions (42.7%) were given for infectious diseases (*p* < 0.001, Table S2). For the other 10 diseases, the proportion of positive opinions ranged from 37.4% to 52.4%, whereas that of negative opinions ranged from 21.7% to 30.6%. Among these 10 diseases, positive opinions exceeding 50% were observed for diabetes (52.4%), cardiovascular disease (51.2%), and cerebrovascular disease (50.2%). Fewer positive opinions were observed for mental illnesses, such as autism (39.7%), schizophrenia (38.5%), and depression (37.4%). The difference between positive and negative opinions was 70.9% for cancer. For most of the adult-onset diseases, there was a difference of 20% or more between positive and negative opinions (diabetes, 27.9%; heart disease, 29.4%; cerebrovascular disease, 28.3%; allergic disease, 24.5%; dementia, 20.9%; and aneurysm, 23.5%), and the number of positive opinions was higher. For mental illnesses with fewer positive opinions, the positive opinions still outnumbered the negative opinions; however, the difference was approximately only 10% (autism, 13.4%; schizophrenia, 9.1%; and depression, 6.7%). The only disease for which the negative opinions outnumbered the positive opinions was infectious diseases, with a difference of 18.6%. Compared to physicians, others (nurses, pharmacists, and registered dieticians) provided significantly more positive opinions for all diseases (*p* < 0.05), and both groups showed a trend toward the most positive opinions for cancer and the least positive opinions for infectious diseases (Figure 2).

### 3.3. Awareness of the Appropriate Age for Genetic Testing

**Figure 3:**
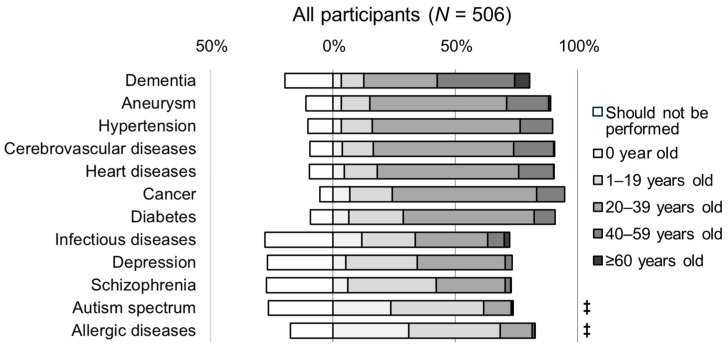
Perceptions of the appropriate age for genetic testing for risk prediction of multifactorial diseases. Statistical significance by pairwise comparisons of proportions with Bonferroni correction between an age below 20 years and an age of 20 years or older, except “should not be performed”, comparing 12 diseases: ‡ *p* < 0.001.

The responses regarding the appropriate age for PRSs are shown in Figure 3. Diseases for which more than 50% of the responses indicated an age of 20 years or older (20–39 years, 40–59 years, ≥60 years) were dementia (67.8%), cerebral aneurysm (73.9%), hypertension (73.7%), cerebrovascular disease (74.1%), heart disease (72.1%), cancer (70.4%), and diabetes (62.1%). Among these listed diseases, for all conditions except dementia, the 20–39 years category was indicated by more than 50% of the responses, ranging from 55.9% to 60.5%. For dementia, the highest number of responses indicated the 40–59 years age group (31.8%). In contrast, for allergies and autism spectrum disorder, the proportion of responses indicating an age below 20 years (0 years and 1–19 years) exceeded 50% (68.4% and 61.7%, respectively, *p* < 0.001, Figure 3 and Table S3). In addition, for infectious diseases and psychiatric disorders, a relatively high proportion of responses (approximately 30%) indicated “should not be performed”, (27.9% for infectious diseases, 26.9% for depression, 27.3% for schizophrenia, and 26.5% for autism spectrum disorder).

**Figure 4:**
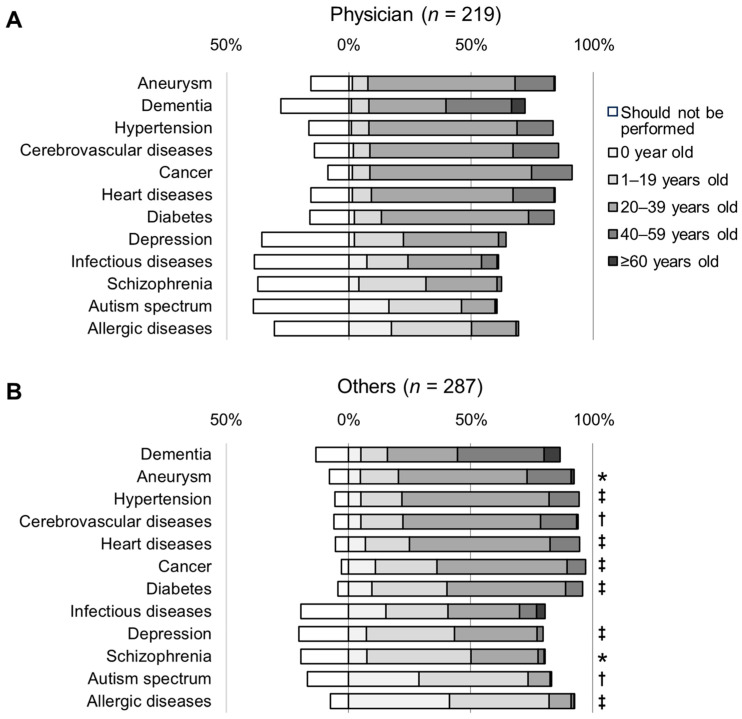
Perceptions of the appropriate age for genetic testing for risk prediction of multifactorial diseases. (**A**) Physicians. (**B**) Others (nurses, pharmacists, and registered dieticians). Statistical significance by Wilcoxon rank sum test compared to physicians’ perceptions: * *p* < 0.05; † *p* < 0.01; ‡ *p* < 0.001.

Compared to physicians, the participants from other categories provided significantly more responses indicating an age below 20 years for 10 diseases, except for infectious diseases and dementia (*p* < 0.05, Figure 4).

### 3.4. Awareness of Diseases Related to Participants’ Practice

**Figure 5:**
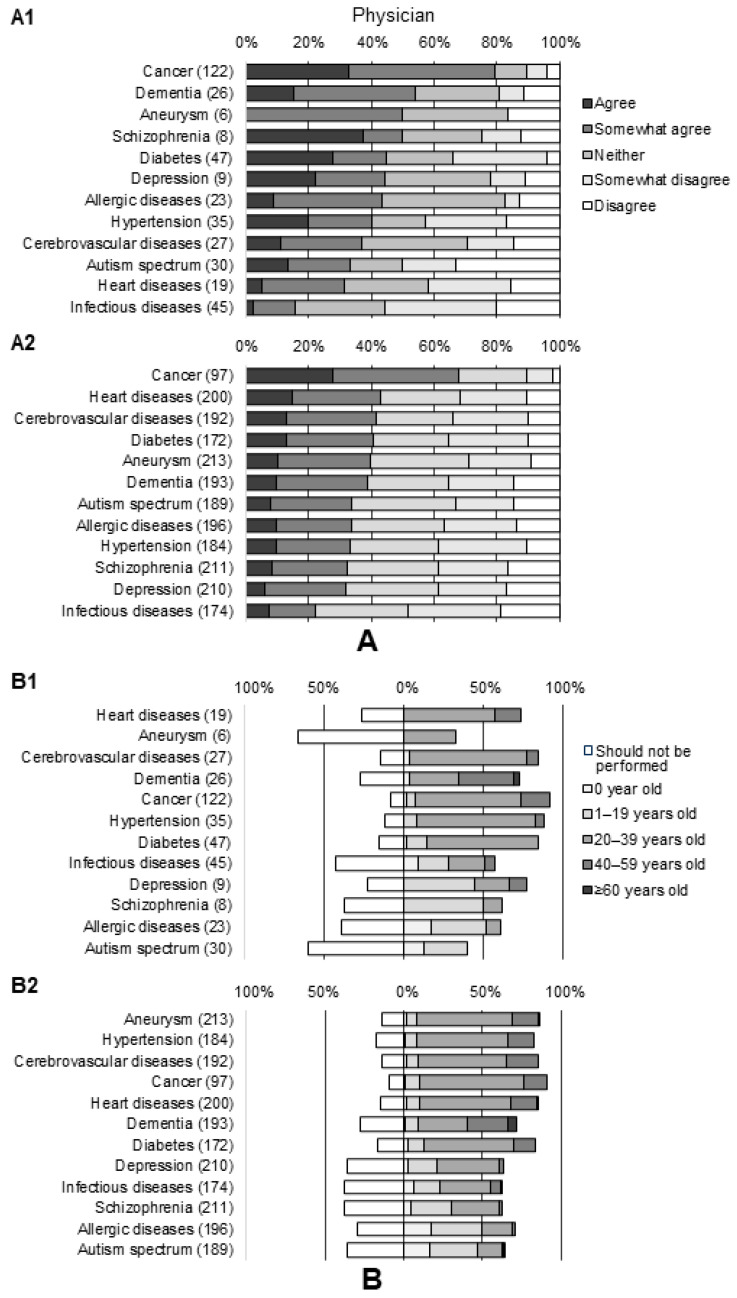
Awareness of diseases related to physicians’ practice. (**A**) Perceptions of the need for genetic testing for multifactorial disease risk prediction. (**B**) Perceptions of the appropriate age for genetic testing for risk prediction of multifactorial diseases. (**A1**,**B1**) Physicians with specialization related to the disease. (**A2**,**B2**) Physicians with specialization not related to the disease.

There was no significant difference between the responses provided by physicians with different specializations, irrespective of whether the disease was related or unrelated to their practice (Figure 5).

Nurses who were not related to the disease provided significantly fewer positive opinions for cerebrovascular diseases and diabetes, and significantly fewer responses indicating an age below 20 years for hypertension and diabetes (Figure S1). Pharmacists who were not related to the disease provided significantly fewer positive opinions for diabetes and depression (Figure S2). Registered dieticians who were not related to the disease provided significantly fewer responses indicating an age below 20 years for heart diseases and aneurysm and more responses indicating an age below 20 years for depression and schizophrenia (Figure S3).

## 4. Discussion

Our research performed a comprehensive survey involving critical stakeholders in personalized preventive medicine, encompassing a wide range of professionals such as physicians, nurses, pharmacists, and registered dietitians. The primary objective of this survey was to assess the level of awareness of these professionals and to ascertain their opinions on the appropriate age for conducting genetic risk prediction for multifactorial diseases. The outcomes of this survey are enlightening, offering a multifaceted perspective on the role of genetic testing in the proactive management of various health conditions.

The participants expressed a generally positive attitude toward the necessity of genetic risk prediction for multifactorial diseases. This positive sentiment was especially evident in the context of diseases such as cancer, diabetes, cardiovascular ailments, allergic reactions, and dementia. The enthusiasm shown for the role of genetic testing in these diseases indicates a growing recognition of its potential benefits for predicting and managing these health conditions. It also suggests an increased understanding among healthcare professionals of the genetic underpinnings of these diseases and the role that personalized medicine can play in their management.

However, the survey also revealed a more reserved stance towards genetic testing in the context of mental and infectious diseases. The observed caution in the healthcare community, as indicated by the results of this survey, highlights an area of potential concern and ambiguity regarding the application of genetic testing for mental and infectious diseases. The fewer positive opinions in these areas may reflect the complexity and sensitivity surrounding diagnosing and managing mental and infectious diseases, suggesting a need for further education and research in these fields.

Regarding the appropriate age for genetic testing, the survey indicated a consensus among the respondents for adult-onset diseases like dementia, with most respondents agreeing that testing should be conducted at the age of 20 years or older. For diseases such as cancer, cardiovascular disease, and diabetes, a narrower age range of ‘20–39 years’ was deemed suitable. The preference for these age ranges reflects a proactive approach towards the early detection and management of these conditions, aligning with the current trends in preventive healthcare.

Our study is the first survey to explore stakeholders’ perceptions of genetic testing for the risk prediction of multifactorial diseases across various disease domains. Some studies highlighted the clinical validity of PRSs for common cancers, including breast and prostate cancer, and cardiometabolic disorders like coronary artery disease, obesity, diabetes, and Alzheimer’s disease [5,30].

Several surveys have also shed light on the implementation of PRSs, in other words, on the clinical utility of PRSs, in intervention studies involving hundreds to thousands of individuals with heart disease, type 2 diabetes, and cancer [11]. These intervention studies demonstrated the practical applicability of PRSs in real-world settings, offering valuable insights into how genetic testing can guide preventive and therapeutic strategies. The Accelerating Detection of Disease Challenge in the U.K., which aims to establish a genomic cohort of five million individuals, exemplifies the growing emphasis on early detection and intervention for major chronic diseases [12]. The large-scale initiative represented by the Accelerating Detection of Disease Challenge is poised to significantly contribute to our understanding of the genetic underpinnings of such diseases and enhance the effectiveness of early intervention strategies.

In the field of psychiatric disorders, such as depression and schizophrenia, there are high expectations for PRSs, owing to the lack of clinically useful biomarkers to date and of a clear pathophysiological etiology for these conditions. However, because the effect sizes of individual single-nucleotide polymorphisms in the pathogenesis of psychiatric disorders are smaller than in the pathogenesis of chronic diseases [31], larger sample sizes are required to explain the involvement of genetic factors in these diseases. PRSs have not yet achieved sufficient discriminatory power for clinical use in many psychiatric disorders [32,33,34].

In current medical practice, genetic testing of unaffected individuals is considered when future disease onset can be reliably predicted or when pre-onset diagnosis has substantial healthcare benefits [35,36,37]. One reason why genetic testing is approached with caution regards the concerns about the possible social discrimination faced by individuals with a positive diagnosis and the consequent psychological impact. Previous research showed that healthcare providers who are more aware of the existence of discrimination are more likely to refrain from offering genetic testing, as reported in the cancer context [38]. Meanwhile, several studies showed that receiving genetic test results for presymptomatic diagnoses of monogenic diseases does not result in harmful psychosocial effects in children and adolescents, highlighting the need for more research into the risks and benefits of genetic testing in childhood [39,40,41]. Although the 12 multifactorial diseases discussed in this study are not necessarily those for which medical intervention should be performed before disease onset, preventive interventions should be provided before the onset of the disease. Given the current general medical practices, PRS implementation in relatively casual settings, such as health checkups, is suggested for the simultaneous risk prediction of several diseases. It would be necessary to reevaluate the potential benefits of disease-specific genetic testing in the context of preventive medicine.

In this study, the stakeholders recognized the importance of genetic risk testing for diseases that develop in adulthood, such as cancer, cardiovascular disease, diabetes, and dementia. They agreed that the appropriate age for testing should be post maturity. Although there were reservations about testing for psychiatric disorders, most respondents supported testing at or before the age of 19 years, ideally before the onset of the disease. This approach requires careful consideration owing to the potential psychosocial implications of predicting the risk of developing mental illnesses, including impacts on personal identity, discrimination, and concerns over the control of personal information [9,42,43].

In this study, physicians showed no significant difference in disease awareness based on their specialty. However, nurses, pharmacists, and registered dieticians not specializing in certain diseases showed varied levels of awareness and opinions. The level of awareness and perception among healthcare professionals about the need for genetic risk testing for multifactorial diseases varied based on their relation to the disease. This finding is crucial from the perspective of implementing genetic risk testing for the examined 12 multifactorial diseases. The differing perceptions and awareness levels might impact the adoption and advocacy of genetic testing across various healthcare sectors [44]. It highlights the need for targeted education and training programs for healthcare professionals tailored to address these perceptual gaps and enhance the overall effectiveness of genetic risk testing implementation in healthcare practices.

In this study, recognition of the need for the genetic risk testing for adult-onset chronic diseases, such as cancer, cardiovascular disease, diabetes, and dementia, was observed. Therefore, these findings can serve as a basis for establishing the practical application of PRSs in clinical settings for determining the risk of multifactorial disease development. However, when discussing clinical implementation, it is important to consider the pathology and age of disease onset. We observed distinct variations in participant characteristics across different occupations. Participants’ diverse demographic and professional backgrounds underscore the importance of considering these varied perspectives when implementing genetic risk testing in clinical settings. Hence, future research should be aimed at identifying implementation barriers and facilitators. Moreover, this study has a limitation; the participants were not prompted to discuss the reason for answering “should not be performed.” Therefore, further research that addresses this limitation is required.

## Figures and Tables

**Table 1:** Participant characteristics (*N* = 506).

Characteristic		Number (%)
Gender		
	Female	267 (52.8)
	Male	224 (44.3)
	Do not wish to disclose	15 (3.0)
Profession		
	Physician	219 (43.3)
	Nurse	112 (22.1)
	Pharmacist	107 (21.1)
	Registered dietitian	68 (13.4)
Age		
	20–29	74 (14.6)
	30–39	167 (33.0)
	40–49	158 (31.2)
	50–59	76 (15.0)
	60–69	30 (5.9)
	≥70	1 (0.2)
Years of experience		
	0–10	177 (35.0)
	11–20	165 (32.6)
	21–30	118 (23.3)
	31–40	40 (7.9)
	≥41	6 (1.2)
Diseases related to their practice (multiple answers)		
	Cancer	308 (60.9)
	Diabetes	195 (38.5)
	Hypertension	167 (33.0)
	Heart diseases	131 (25.9)
	Infectious diseases	120 (23.7)
	Cerebrovascular diseases	93 (18.4)
	Dementia	77 (15.2)
	Allergic diseases	73 (14.4)
	Depression	59 (11.7)
	Schizophrenia	50 (9.9)
	Autism spectrum	46 (9.1)
	Aneurysm	32 (6.3)
	Not applicable	48 (9.5)

## Data Availability

The datasets used and analyzed in the current study are available from the corresponding author upon reasonable request. The data are not publicly available due to privacy protection concerns.

## Supplementary Materials

The following supporting information can be downloaded at: https://www.mdpi.com/article/10.3390/genes15010049/s1, Figure S1: Awareness of diseases related to nurses’ practice; Figure S2: Awareness of diseases related to pharmacists’ practice; Figure S3: Awareness of diseases related to registered dietitians’ practice; Table S1: Participant characteristics (physicians and others); Table S2: Pairwise comparison of the necessity of genetic testing between diseases; Table S3: Pairwise comparison of the appropriate ages to perform genetic testing between diseases.
