# Supplementary material for: Stakeholder Perception of the Implementation of Genetic Risk Testing for Twelve Multifactorial Diseases
Source: Genes (Basel). 2023 Dec 28;15(1):49. doi: 10.3390/genes15010049 (PMC10815213; doi:10.3390/genes15010049)
Supplement: Supplementary file 1 [file genes-15-00049-s001.zip › Figure_S2.pptx]

## Slide 1
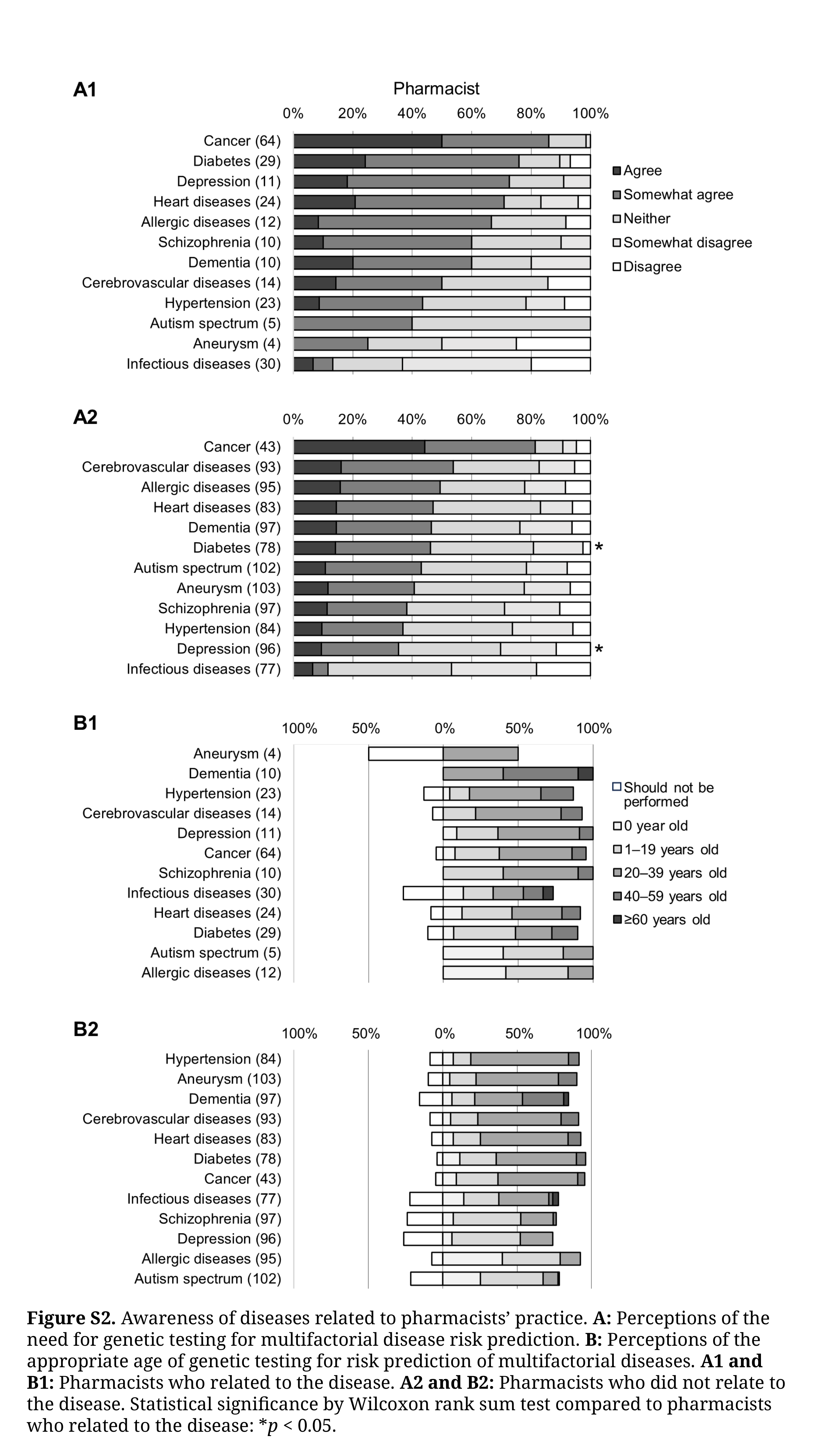

Figure S2. Awareness of diseases related to pharmacists’ practice. A: Perceptions of the need for genetic testing for multifactorial disease risk prediction. B: Perceptions of the appropriate age of genetic testing for risk prediction of multifactorial diseases. A1 and B1: Pharmacists who related to the disease. A2 and B2: Pharmacists who did not relate to the disease. Statistical significance by Wilcoxon rank sum test compared to pharmacists who related to the disease: *p < 0.05.
